# Vitamin D: Dosing, levels, form, and route of administration: Does one approach fit all?

**DOI:** 10.1007/s11154-021-09693-7

**Published:** 2021-12-23

**Authors:** John P. Bilezikian, Anna Maria Formenti, Robert A. Adler, Neil Binkley, Roger Bouillon, Marise Lazaretti-Castro, Claudio Marcocci, Nicola Napoli, Rene Rizzoli, Andrea Giustina

**Affiliations:** 1 Department of Medicine, Endocrinology Division, Vagelos College of Physicians and Surgeons, Columbia University, New York, NY USA; 2 Institute of Endocrine and Metabolic Sciences, San Raffaele, Vita-Salute University and IRCCS Hospital, Milano, Italy; 3 McGuire Veterans Affairs Medical Center and Virginia Commonwealth University School of Medicine, Richmond, VA USA; 4 University of Wisconsin, Madison, WI USA; 5 Laboratory of Clinical and Experimental Endocrinology, Department of chronic diseases, metabolism and ageing, Leuven, KU Belgium; 6 Division of Endocrinology, Escola Paulista de Medicina – Universidade Federal de Sao Paulo (EPM-UNIFESP), Sao Paulo, Brazil; 7 Department of Clinical and Experimental Medicine, University of Pisa, Pisa, Italy; 8 Unit of Endocrinology and Diabetes, Campus Bio-Medico University of Rome, Rome, Italy; 9 Service of Bone Diseases, Geneva University Hospitals and Faculty of Medicine, Geneva, Switzerland

**Keywords:** Vitamin D, Bone, COVID-19, Extraskeletal effects, Obesity, Parathyroid hormone

## Abstract

The 4^th^ International Conference on Controversies in Vitamin D was held as a virtual meeting in September, 2020, gathering together leading international scientific and medical experts in vitamin D. Since vitamin D has a crucial role in skeletal and extra-skeletal systems, the aim of the Conference was to discuss improved management of vitamin D dosing, therapeutic levels and form or route of administration in the general population and in different clinical conditions. A tailored approach, based on the specific mechanisms underlying vitamin D deficiency in different diseases that were discussed, was recommended. Specifically, in comparison to healthy populations, higher levels of vitamin D and greater amounts of vitamin D were deemed necessary in osteoporosis, diabetes mellitus, obesity (particularly after bariatric surgery), and in those treated with glucocorticoids. Emerging and still open issues were related to target vitamin D levels and the role of vitamin D supplementation in COVID-19 since low vitamin D may predispose to SARS-CoV-2 infection and to worse COVID-19 outcomes. Finally, whereas oral daily cholecalciferol appears to be the preferred choice for vitamin D supplementation in the general population, and in most clinical conditions, active vitamin D analogs may be indicated in patients with hypoparathyroidism and severe kidney and liver insufficiency. Parenteral vitamin D administration could be helpful in malabsorption syndromes or in states of vitamin D resistance.

Specific guidelines for desired levels of vitamin D should be tailored to the different conditions affecting vitamin D metabolism with the goal to define disease-specific normative values.

## Introduction

**Fig. 1:**
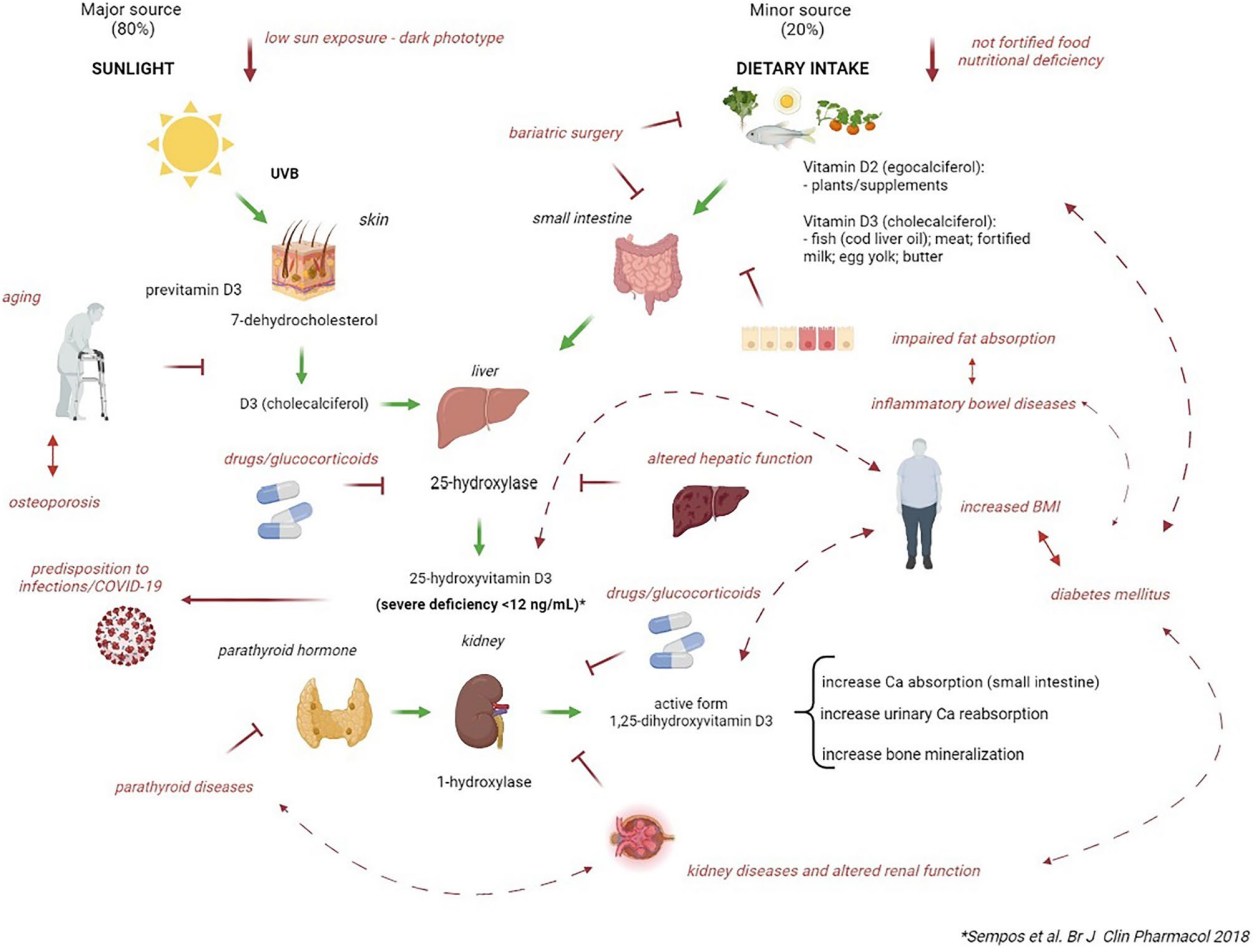
Vitamin D metabolism and its alterations in different clinical conditions (Adapted from Ref 115)

The 4^th^ International Conference on Controversies in vitamin D was held virtually in September, 2020. It followed three previous meetings in 2017 [1], 2018 [2] and (2019) [3] which convened leading international experts in vitamin D to address ongoing controversies and timely issues related to vitamin D. Formal presentations on specific topics were followed by discussion among experts to help identify lingering issues and to clarify areas of uncertainty. Based on the evidence that vitamin D, besides its vital importance for the skeleton, may have multiple extra-skeletal effects [4], the main aim of the Conference was to discuss if the paradigm of a standard vitamin D dose, therapeutic level and form or route of administration was still acceptable or if a tailored approach based on the specific pathophysiology of the altered vitamin D metabolism in the different clinical conditions, which will be highlighted in the first section of the paper and summarized in Fig. 1 could be identified and recommended. To reach this objective, the authors focused their discussion on the following topics which will be summarized in the different sections of the current manuscript: 1. The amount of vitamin D to reach a desired level varies in the general population vs subjects with different clinical conditions such as obesity, inflammatory bowel diseases (IBD), parathyroid diseases or treated either with bariatric surgery or glucocorticoids; 2. how and whether optimized serum 25-hydroxyvitamin D [25(OH)D] concentrations vary by disease states in which vitamin D is implicated such as metabolic bone diseases, diabetes mellitus, gastrointestinal, kidney, and neurological diseases as well as malignant disorders and infections. In this regard, a new area reviewed was the role of vitamin D in SARS-CoV-2 infection and how it may relate to COVID-19 outcome [5–8]; 3. Indication to use the available forms and regimens of vitamin D supplementation according to the disease underlying vitamin D deficiency were discussed. In particular, the role of cholecalciferol vs ergocalciferol and active vitamin D analogs was discussed. Several conditions were identified in which active vitamin D analogs were indicated such as hypoparathyroidism and severe kidney and liver insufficiency. Finally, possible role of parenteral vitamin D administration in malabsorption syndromes was discussed.

## Vitamin D metabolism and assessment of vitamin D status

Vitamin D is essential for intestinal calcium absorption and for the prevention of rickets or osteomalacia. It also plays in role in calcium, phosphate, and bone metabolism in general besides having multiple extra-skeletal effects [4].

Vitamin D is a fat-soluble secosteroid produced by the skin after exposure to sunlight. The photolytic reaction is induced by irradiation of 7-dehydrocholesterol (pro-vitamin D) in the skin, by an energetic UV-B wavelength of approximately 280 to 310 nm) [3, 9] (Fig. 1).

UV-B irradiation of 7-dehydrocholesterol promotes a photochemical cleavage between carbons 9-10 of the steroid ring generating the pre-vitamin D3 hormone. Through a time (up to 48 hours) and temperature-dependent molecular isomerization, pre-vitamin D2 is converted to vitamin D3 (cholecalciferol), as well as to two biologically inert products, tachysterol and luminosterol. Dermal synthesis of vitamin D depends upon the intensity of UV radiation (related to time of day, latitude, season) and air pollution [9].

Dietary intake does not provide more than 20% of the daily vitamin D requirement with the major dietary sources being dairy and cereal products, mushrooms, egg yolks and fish oils. Plants are also a source of vitamin D in the form of vitamin D2 (ergocalciferol), an equivalent biologic form. Dietary vitamin D is initially absorbed in the small intestine, bound to chylomicrons where it is transported to the lymphatic vessels and thereafter enters the circulation bound to vitamin D binding protein (VDBP) [10]. The biologically active form of vitamin D is obtained through sequential hydroxylation steps in the liver and the kidneys.

The first hydroxylation step at C-25 (carbon atom-25) occurs in the liver by the vitamin D-25-hydroxylase (CYP2R1), a mitochondrial cytochrome P450–like enzyme, converting cholecalciferol into 25(OH)D). The half-life of 25(OH) D is approximately 2-3 weeks. The final hydroxylation step occurs in the renal proximal convoluted tubules by the vitamin D-1-hydroxylase (CYP27B1), a cytochrome P450–like oxidase, converting 25(OH)D to 1,25-dihydroxycholecalciferol (calcitriol), the active form of vitamin D. The half-life of calcitriol is very short, approximately 6-8 hours. [9]

Both 25(OH)D and calcitriol can also be hydroxylated by the vitamin D-24-hydroxylase (CYP24A1), present in several human different tissues, generating the 24,25(OH)2D3 and the 1,24,25(OH)3D3, vitamin D metabolites that do not have any major biological role. These forms of vitamin D are excreted in bile, feces, and urine [11].

Since, as reported above, most vitamin D comes from synthesis in the skin, the elderly and those institutionalized and/or individuals with dark skin are at higher risk for vitamin D deficiency [3]. To date, the precise amount of UV-B light needed to generate a defined amount of vitamin D or serum 25(OH)D is still unclear and may well differ among individuals [4]. Indeed, divergent studies report that a full day’s body exposure to sunlight may generate about 800 or more than 20,000 IU. Between 34% and 40 % of the US, European and African populations, are living with serum 25(OH)D concentrations classified as deficient (below 20 ng/mL) and either need more sun exposure or greater vitamin D intake [12]. The 1α-hydroxylase is regulated, in part, by parathyroid Hormone (PTH), serum phosphate, calcium and the Fibroblast Growth Factor-23 (FGF-23) [13, 14]. PTH and hypophosphatemia stimulate the action of this enzyme, whereas hypercalcemia and FGF-23 repress it [15]. In contrast to 1α-hydroxylase, the regulation of 25-hydroxylase is poorly understood.

The circulating concentration of 25(OH)D is widely accepted as the best marker of vitamin D status. It is used to establish the appropriate vitamin D dietary and therapeutic requirements in those with vitamin D insufficiency or deficiency [3]. Despite the clinical utility of the 25(OH)D measurement, other forms of vitamin D loom as potentially important physiological indices such as free unbound 25(OH)D, and the ratio between 24,25(OH)2D/25(OH)D, the latter being important in disorders such as idiopathic infantile hypercalcemia, and in states of altered CYP27B1 enzyme activity [16, 17]. These forms of circulating analogues, however, have not shown themselves to be useful, except as noted above, because laboratory measurement technology is not uniformly reliable. [18, 19].

We are left with 25(OH)D, even better than measurement of 1,25(OH)2 D, as the best index of vitamin D stores. Values below 12 ng/mL (30 nmol/L) are widely considered to be associated with an increased risk of rickets/osteomalacia. Levels of 25(OH)D between 12 and 20 ng/mL are considered to be in the insufficient range. Levels between 20 ng/mL and 50 ng/mL (50–125 nmol/L) appear to be safe and sufficient for the general healthy population [20].

The amount of oral vitamin D needed to prevent rickets or secondary hyperparathyroidism has been studied in many observational and in a few randomized controlled trials (RCTs) over the last 100 years. A daily intake of 200-400 IU of vitamin D (5-10 ug) is sufficient to prevent rickets in infants and to bring serum 25(OH)D above the minimal threshold of 12 ng/mL (30 nmol/L.[3],

Many studies have evaluated serum 25(OH)D concentrations after vitamin D supplementation of children, adolescents, adults, and elderly subjects. There is consensus that the increase in serum 25(OH)D depends on the baseline vitamin D status as the change in 25(OH)D, for a given amount of vitamin D, is highest in vitamin D deficient subjects and gradually diminishes in subjects who are more replete. Moreover, the amount of vitamin D needed to reach a given value, such as 20 ng/mL, depends on age, body weight, gene polymorphisms and assay methodology [21–23]. Available studies are also handicapped by compliance problems. Nevertheless, the amount of vitamin D needed to bring the 25(OH)D level up to 20 ng/mL requires a daily dose between 600 and 1,800 IU/d for virtually all age groups [24].

## The amount of vitamin D to reach a desired level varies according to the clinical conditions

- **Obesity**
  The association between hypovitaminosis D and overweight/obesity is widely recognized [25–27]. Several pathophysiological mechanisms have been hypothesized to explain this association. Excess body fat can serve as a repository of vitamin D and, thus, alter the kinetics between that depot and the circulation. In addition, obesity may be associated with lower dietary intake of vitamin D, reduced outdoor physical activity with limited skin exposure to sunlight, impaired hydroxylation in adipose tissue, and alterations in vitamin D receptors. [25–27].
  Because the vitamin D receptor (VDR) is expressed on adipocytes, it is intriguing to consider the possibility that vitamin D may play a role in modulating adipose tissue distribution and function [28]. Obese subjects, particularly if elderly, often demonstrate low levels of 25(OH)D, which are inversely correlated with body mass index and adiposity [29, 30], influencing negatively skeletal and muscle health with a resulting increased predisposition to obese osteo-sarcopenic phenotype [31, 32]. Prevalence of vitamin D deficiency is 35% and 24% higher in obese and overweight persons, respectively, than in normal-weight subjects [29]. With adipose tissue serving as a storage organ, low circulating levels could reflect sequestration of vitamin D in this site [29, 30]. The mechanism by which vitamin D is stored in adipose tissue has not been elucidated.
  Another consideration is the point that overweight and obese subjects are resistant to vitamin D supplementation, when compared to their lean counterparts. In fact, a recent meta-analysis showed that after administration of equal doses of vitamin D in obese subjects, serum 25(OH)D were lower by about 15.2 ng/mL (38 nmol/L). [33]. Another recent study showed that in obese children receiving an equivalent dose of vitamin D [34], circulating levels are 45% lower. How or whether VDRs on adipocytes may account for these differences is not clear [16, 17].
  Many studies have reported that 1,25(OH)2D regulates adipocyte differentiation by binding to the nuclear VDR with high affinity. How this observation translates clinically with regard to regulation of adipogenesis when vitamin D is administered is uncertain [35, 36]. Vitamin D appears also to affect energy metabolism in several different ways although evidence is limited to animal models. [37]. *In vivo* animal studies have consistently demonstrated that vitamin D regulates the recruitment of immune cells into adipose tissue but clinical evidence is lacking [38]. It is apparent that more studies are needed to clarify the role of vitamin D on adipose tissue and on obesity.
  In the last two years, higher BMI and altered body composition with increased adiposity were reported as independent risk factors for greater disease severity and poor prognosis in inflammatory and infectious disease such as COVID-19 [39, 40]. A recent study, aiming to evaluate the possible relationship between BMI and vitamin D in COVID-19 patients, observed a negative relationship between higher BMI and lower vitamin D levels and reported that overweight patients and hypovitaminosis D were affected by more severe disease with worse inflammatory parameters and poor outcomes compared to those with one or none of these two conditions [41]. These data suggest that vitamin D may exert a protective effect in obese individuals by reducing systemic inflammation in these patients [28]. Moreover, since vitamin D has been suggested to play a role in modulating fat distribution and activity, adequate vitamin D status may also be key in preserving body composition in the post COVID-19 recovery period [42].
- **Bariatric surgery**
  The most widely used bariatric surgery procedures are the sleeve gastrectomy (SG) and Roux-en Y gastric bypass (RYGB). With SG, more than 80% of the stomach is transected, causing a rapid transit of nutrients through the gastric outlet [43]. The range of Vitamin D deficiency after SG is wide, between 14 and 72 %, 1year post-surgery. Vitamin D daily doses of at least 3000 IU have been used in order to reach a threshold of 28 ng/mL (70 nmol/L) [44].
  RYGB has both restrictive and malabsorptive features and leads to substantial weight loss in morbidly obese patients [45]. The long-term positive effects on weight as well as diabetes [45] prevention is potentially offset by bone loss and higher fracture risk [46]. In fact, RYGB affects bone metabolism through mechanical unloading, hormonal and bone marrow fat modifications, and deficiencies in vitamin D, calcium and other nutrients. It is likely that the malabsorption induced by the surgical bypass procedure modifies the delivery of pancreatic secretions and bile salts leading to reduced vitamin D absorption. In fact, low levels of vitamin D are common after RYGB. Doses up to 5,000 IU per day have stabilized levels in these subjects since supplementation with usual doses of vitamin D (800 IU daily) had a limited effect on preventing vitamin D deficiency [47].
  A less common surgical procedure is the biliopancreatic diversion that causes massive malabsorption of minerals and fat-soluble vitamins. Vitamin D deficiency and increased PTH levels are common findings in patients undergoing biliopancreatic diversion (ranging from 60 to 100% according to different studies) [48].
  The consensus is that high doses of vitamin D supplementation and, perhaps, intramuscular vitamin Dadministration should be considered in patients who have undergone bariatric surgery. Postoperative maintenance of adequate vitamin D levels is crucial in order to prevent bone loss and to maintain bone health. Additional prospective studies and clinical guidelines are needed to determine how to optimize vitamin D nutrition and avoid possible skeletal complications following GB.
- **Exogenous and endogenous glucocorticoid excess**
  A meta-analysis of observational studies has found that most adult subjects administered pharmacological amounts of glucocorticoids had low 25(OH)D levels, irrespective of what threshold value was used to define hypovitaminosis D [49]. The bone loss associated with glucocorticoids [50] include distorted PTH pulsatility [51] and pathophysiological perturbations in vitamin D synthesis, metabolism, and action. Interference with vitamin D synthesis occurs at separate steps leading to impaired production of active vitamin D. Vitamin D metabolism, furthermore, is heavily influenced by chronic exposure to either endogenous or exogenous glucocorticoid excess. Glucocorticoids are also associated with target tissue resistance to vitamin D [52].
  It is, therefore, important to consider vitamin D supplementation when patients are exposed to glucocorticoid excess [53]. Therapeutic goals should take into account two particular points [54]. Resistance to vitamin D dictates a higher level of 25(OH)D level to ≥ 32 ng/mL. Daily administration of 2,000 IU often will reach and maintain this goal [55]. A second therapeutic point relates to synthetic interference with production of active vitamin D, necessitating in some situations the use of active forms of vitamin D, such as calcidiol or calcitriol [56].
- **Gastrointestinal diseases**
  Vitamin D availability is important in regulating gut mucosal immunity [56, 57]. Vitamin D deficiency is common in the two major forms of inflammatory bowel disease (IBD), namely Crohn’s disease (CD) and ulcerative colitis (UC). Several mechanisms contribute to vitamin D deficiency in IBD. These include impaired absorption of nutrients and bile salts, dietary restriction, lack of sun exposure and treatment with glucocorticoids [58, 59].
  In a retrospective cohort study among 504 IBD patients, Ulitsky et al. found that vitamin D deficiency was common among CD patients and was independently associated with greater disease activity [60]. In addition, a cross-sectional study by Rafferty et al. showed among 199 CD patients that circulating 25(OH)D was inversely associated with markers of intestinal inflammation [61]. In 711 CD and 764 UC Korean patients, reduced 25(OH)D levels were associated with higher disease activity scores and CRP levels (p < 0.001). 25(OH)D levels <10 ng/mL were found to be associated with ileocolonic disease and complicated CD or disease extent and CMV colitis in UC (p < 0.001). Additionally, severe 25(OH)D deficiency was associated with CMV colitis. In a multivariable analysis, severe deficiency of 25(OH)D was an independent risk factor for surgery in both CD and UC [62].
  In a retrospective study, more than half of 83 pediatric IBD (mostly CD) patients had 25(OH)D levels <30 ng/mL. In CD, proximal gastrointestinal tract inflammation and early termination of anti-TNF therapy were associated with vitamin D insufficiency [63].
  Among a cohort of 89 CD patients, Yamada et al. found that 17 (19.1%), 46 (51.6%), and 26 (29.2%) patients had 25(OH)D levels of <15, 15-30, and >30 ng/mL, respectively. Patients with higher vitamin D levels were significantly more likely to be in endoscopic remission than those with lower levels. On multivariate analysis, vitamin D levels >30 ng/mL (odds ratio [OR] 0.22, 95% confidence interval [CI] 0.07-0.66, p = 0.006) and anti-tumor necrosis factor agent treatment (OR 0.25, 95% CI 0.08-0.83, p = 0.01) were associated with reduced risk of endoscopic recurrence [64].
  Jorgensen et al. evaluated the effect of oral vitamin D3 treatment in a double-blind placebo-controlled trial of 94 CD patients randomized to receive either placebo or 1,200 IU/d for 12months. The relapse rate was reduced from 29% in the placebo group to 13% in the treatment group [65]. Another double-blind randomized placebo-controlled study showed that short-term treatment with 2,000 IU/day Vitamin D significantly increased 25(OH)D levels in CD patients, enhanced circulating levels of the antimicrobial peptide LL-37, and maintained intestinal permeability [66].
  In a recent randomized study, it has been observed that patients with CD not treated with vitamin D had greater needs for infliximab dose escalation during follow-up than those treated with vitamin D [67]. These data suggest that vitamin D treatment plays an important role in the pathogenesis and clinical outcomes in IBD.
- **Primary Hyperparathyroidism**
  In primary hyperparathyroidism (PHPT), total serum 25(OH)D concentration is lower as compared to healthy controls [68, 69]. Although the mechanisms accounting for this finding are not clear, several hypotheses have been proposed. 1. patients with PHPT have lower levels of VDBP than controls [70, 71]; 2. PTH enhances the conversion of 25(OH)D to 1,25(OH)2D by inducing the renal 1-alpha hydroxylase enzyme (CYP27B1). It is unlikely that this latter mechanism is relevant because the serum concentration of 25(OH)D is one order of magnitude higher than that of 1,25(OH)2D [72] 3. increased serum 1,25(OH)2D levels inhibit the production of its precursors in the skin and the liver, favoring the downregulation of renal 1-alpha hydroxylase [73]. 4. increased metabolic clearance rate of 25(OH)D is suggested by accelerated fecal loss that is reversible after parathyroidectomy [74]. 5. PTH and 1,25(OH)2D increase the conversion of 25(OH)D to 24,25(OH)2D by activating the kidney expression of CYP24A1 [75]. Despite the lower levels of total 25(OH)D levels in PHPT as compared to healthy controls, there is no difference in serum free and bioavailable levels of 25(OH)D [70, 71]. Thus, it is conceivable that the vitamin D status in PHPT patients is not completely represented by serum 25(OH)D levels. Conversely, in patients with normocalcemic PHPT, free 25(OH)D levels are lower compared to healthy controls. It was postulated that some normocalcemic PHPT subjects have secondary hyperparathyroidism based on their free 25(OH)D levels [75, 76]. 

## Different disorders require different circulating levels of 25(OH)D for optimized outcomes

- **Nutritional vitamin D deficiency**
  Impaired mineralization of the growth plate and increased osteoid surface result in rickets and osteomalacia, respectively. Insufficient sun exposure, low vitamin D intake and/or calcium and phosphate deficiency are the leading causes of nutritional rickets and osteomalacia [77]. With decreasing circulating levels of 25(OH)D below 20-24 ng/mL, there is a concentration-dependent increase in non-vertebral fractures (including hip), cardiovascular events, impairment in muscle function, infections, frailty, and mortality [77, 78]. Bone turnover markers and PTH are highest at 25(OH)D values lower than 10-12 ng/mL, with a progressive decrease up to 20 ng/mL and then a trend to a plateau at values greater than 30 ng/mL [79]. But there is great variability among studies [79]. In a post-mortem study of iliac crest bone biopsies in 675 patients, individuals with serum 25(OH)D levels lower than 10-12ng/mL had greater osteoid volume, surface, and thickness than those with higher 25(OH)D levels [80]. There appears to be little additional difference in osteoid parameters at serum 25(OH)D levels higher than 20 ng/mL. [80].
- **Primary Hyperparathyroidism: to control PTH level**
  In a meta-analysis of 10 studies including 340 patients with PHPT, there was no significant worsening of hypercalcemia following vitamin D supplementation, with only 2.2% of patients developing serum calcium above 12 mg/dL [81]. In most studies, cholecalciferol and ergocalciferol were used, with significant heterogeneity across them. Doses ranged from 800 IU daily to 50,000 IU twice weekly. Serum PTH decreased on average by 33% (p=0.003) [81]. Adequate levels of vitamin D in PHPT will help to control any tendency for PTH levels to rise further, if 25(OH)D levels fall below a threshold value. The concern is that the pathophysiological processes leading the overproduction of PTH in PHPT could be worsened by a secondary stimulation of PTH by virtue of inadequate 25(OH)D levels. The controversial issue of a threshold value for 25(OH)D relates not only to PHPT but also to subjects without known metabolic bone disease [82]. With specific reference to PHPT, Walker et al. have shown that levels below 30 ng/mL are associated with increases in PTH [83]. It has also been shown that increases in 25(OH)D up to 30 ng/mL are associated with a reduction in PTH levels. This observation was also made specifically in PHPT by the metanalysis by Song et al. [81]. The safe use of vitamin D supplementation is also relevant. In individual studies such as the one by Rolighed L et al. vitamin D up to 2,800 IU per day was shown to be safe and associated with reductions in serum PTH and CTX without any changes in serum or urine calcium or serum creatinine [84]. Furthermore, meta-analyses by Loh et al. and by Song et al. concurred that over a range of Vitamin D concentrations, serum calcium and urine calcium excretion were stable [81, 85]. Despite the complex relationship between vitamin D and PTH [86], it seems prudent, based upon the evidence, to recommend levels of 25(OH)D >30 ng/mL in PHPT. Upper limits of 25(OH)D should follow usual nutritional guidelines [1–3].
- **Hypoparathyroidism (Impaired conversion of 25(OH)D to 1,25(OD)D2)**
  Different mechanisms contribute to the impaired conversion of 25(OH)D to 1,25(OH)2D in patients with hypoparathyroidism. First, low levels of PTH reduce the expression and the activity of renal 1-alpha hydroxylase enzyme (*cyp27b1*) [17]. Additionally, the increased serum phosphate level contributes to the downregulation of the *cyp27b1* enzyme directly and, indirectly, by increasing the serum levels of FGF23 [87]. In human subjects, different studies have shown that patients with HypoPT have increased levels of FGF23, as a consequence of the increased levels of phosphate [87]. In this regard, studies in mice showed that FGF23 reduced the expression of *cyp27b1* and stimulated the expression of *cyp24a1*, in a finely balanced homeostatic mechanism Thus, it is conceivable that the high levels of FGF23 may contribute to the impaired conversion of serum 25(OH)D in 1,25(OH)D in patients with hypoparathyroidism [14, 88]. 
- **Osteoporosis**
  Osteoporosis is the most common metabolic bone disorder in adults. Vitamin D with calcium has been tested in multiple studies with many viewing these studies as nutritional supplementation. In most studies of osteoporosis, pharmacologic treatment has included modest nutritional supplements of calcium and vitamin D [89, 90]. There have been several meta-analyses of the impact on fracture risk of calcium and vitamin D, sometimes with different conclusions. In one widely cited meta-analysis [91], this combined supplementation led to an overall 15% decrease in fracture risk. Both the United Kingdom [92] and Endocrine Society [93] osteoporosis guidelines recommend vitamin D as part of a comprehensive approach to osteoporosis treatment, with the UK Guideline specifying at least 20 micrograms (800 IU) daily. Some studies of bisphosphonate therapy [94] have concluded that the serum 25(OH)D level needs to be at least 30 ng/mL (75 nmol/L) in order to gain maximal impact of the bisphosphonate therapy. Expert consensus and position statements of different medical societies [95, 96] agree that, while 20 ng/mL may be adequate for bone health in the population as a whole, the cushion provided by a level of 30 ng/mL is a reasonable, but still controversial, target in patients with osteoporosis. Daily or weekly administration of cholecalciferol is recommended. Very large, intermittent doses, such as 500,000 IU of vitamin D administered once yearly are not recommended because it led to more falls and fractures compared to control subjects not receiving vitamin D [97].
- **Chronic kidney disease**
  Choosing a target 25(OH)D in CKD is more complicated than in most other disease states. The kidney plays a pivotal role in calcium and phosphate homeostasis by the amounts of calcium, phosphate, and acid excreted. The kidney is a target organ for parathyroid hormone and FGF23. Finally and importantly, circulating 1,25(OH)2D is primarily formed in the kidney despite its local production in other tissues [98]. The choice of an ideal serum 25(OH)D level is determined to some extent by the level of renal dysfunction. The KDIGO Guideline [99] suggests measurement of 25(OH)D as well as calcium, phosphate, and parathyroid hormone at stage CKD3 or worse. Patients with CKD 5 stage are at high risk for fracture. Thus, those individuals at stage CKD 3 or 4 who are likely to have worsening renal function should have 25(OH)D levels that are optimal for bone health. Twenty-30 ng/mL is the goal as provided for by daily adequate cholecalciferol supplements. Care must be taken because vitamin D increases phosphate absorption in the gut, which can exacerbate phosphate retention as renal dysfunction progresses. The standard 25(OH)D level also provides substrate for any local production of active vitamin D. In addition, as renal function declines, the ability to activate 25(OH)D is concomitantly decreased, so that more than reassessment of the serum 25(OH)D is required to optimize mineral metabolism status as the stage of CKD advances [100]. Use of 1 alpha hydroxylated vitamin D preparations and calcitriol in CKD5 is discussed below.
- **Multiple sclerosis**
  In younger adults, multiple sclerosis (MS) is a common cause of disability. Considerable epidemiological evidence indicates that low serum levels of 25(OH)D are associated with increased incidence of MS and in some studies disease progression [101]. Vitamin D receptors are found on neural cells, and the potential impact of vitamin D on immune function are two potential mechanistic pathways for vitamin D to affect MS. There have been many, mostly small studies, of varying doses of vitamin D on disease progression. A recent meta-analysis [102] did not support large doses (up to 40,000 IU daily) of cholecalciferol in MS. An illustrative recent trial [103] found no difference in neurological outcomes in patients receiving 20,400 IU or 400 units daily in addition to interferon-β1b. Instead, for most patients with MS, a serum target of 20-30 ng/mL is reasonable until ongoing studies prove otherwise, using standard doses of daily cholecalciferol [104].
- **Diabetes mellitus**
  Several studies and large clinical trials have investigated the role of vitamin D in relation to type 2 diabetes (T2D) onset and progression. In general, the results have not given clear signals of a relationship, but different vitamin D dosages and study designs make this a complex mixture of results [105]. In contrast, a post hoc analysis of the D2d trial showed that vitamin D was associated with a beneficial effect to reduce the onset of overt diabetes but only in those with vitamin D deficiency at baseline (62% diabetes onset reduction if 25(OH)D <12ng/mL) [106]. In a meta-analysis comprising 4896 subjects, Vitamin D supplementation reduced the risk of T2D in patients with prediabetes (RR 0.89, CI 0.80–0.99] and increased the reversion rate of prediabetes to normoglycemia (RR 1.48, CI1.14–1.9) [107]. A recent meta-analysis did not give evidence that vitamin D improves insulin sensitivity [108].
  During early childhood, vitamin D may decrease type 1 diabetes (T1D) risk [109, 110] although no significant effects were observed on insulin secretion, insulin sensitivity, or insulin requirements in newly diagnosed children with T1D [111, 112]. Higher 25(OH)D levels at birth predicted a lower risk of developing T1D or islet autoimmunity depending on VDR genotype (VDR rs11568820 G/G genotype [113] and VDR rs7975232 [114] respectively. Both children or maternal VDR SNPs may lower VDR expression and, by consequence, inhibit T-cell proliferation, and increasing risk of autoimmunity.
  Finally, it is interesting to note that patients with circulating vitamin D levels below 20 or even 30 ng/mL are at higher risk of developing diabetic microvascular complications and in particular diabetic retinopathy and its severity [115].
- **Malignancy**
  Since the Garland brothers' first publication in 1980 [116] showing an association between solar radiation and colon cancer incidence and mortality, the therapeutic potential of vitamin D in cancer has been intensively investigated. At the same time, experimental studies have demonstrated antineoplastic effects of active vitamin D, providing plausibility for the clinical observations [117]. Several systematic reviews have described an association between low levels of 25(OH)D (<20 ng/mL) and higher incidence and mortality for different types of cancer, especially colon and breast cancer [118, 119]. Admittedly, the results have not all been consistent [120]. Following these observations, large RCTs addressing the question of vitamin D and malignancy have met with controversial results [121]. On the other hand, interventional studies have shown that survival data are better while incidence is unchanged with vitamin D supplementation [122]. Explanations for these findings include less aggressive forms of cancer as well as a more favorable response to antineoplastic treatment. A secondary analysis of the VITAL study, a large RTC study that included 25,871 individuals, demonstrated recently that supplementation with vitamin D 2,000 IU daily for 5 years reduced the incidence of metastatic or fatal cancer compared to placebo [HR 0.83; 95%CI 0.69-0.99; p=0.04), with the strongest effect in individuals of normal weight (HR 0.62; 95%CI 0,45-0.86) [123]. These results support others [124–127] and suggest that an apparent protective effect of vitamin D prolonging survival appears to require concentrations of 25(OH)D>30 ng/mL. A recent metanalysis confirmed the effect of higher levels of 25(OH)D on overall survival and progression-free survival [128]. While not conclusive, by any means, a general consensus at this time is that vitamin D has a beneficial effect to reduce cancer mortality.
- **Infections**
  Immune cells have all the machinery for the production and action of active vitamin D. They synthesize the VDR and also have the ability to produce the enzyme 1-alpha hydroxylase (CYP27B1), responsible for converting 25(OH)D to the active metabolite. Thus, for this autocrine action on the immune system, appropriate serum levels of 25(OH)D are important [3]. Active vitamin D regulates the innate immune system, improving the ability of cells to kill pathogenic microorganisms [3]. Serum levels of 25(OH)D are inversely associated with infection risk. In children, pneumonia is more frequent among those with vitamin D deficiency (25-OH D <20 ng/mL) [129]. In adults, when 25(OH)D ranged between 30 and 50 ng/mL, the risk of hospital-acquired infections was reduced [130]. A protective effect of vitamin D supplementation on acute respiratory infections (ARI) was demonstrated in a double-blind RCT in children from Mongolia with very low 25(OH)D baseline levels (RR: 0.41 [95% CI: 0.20– 0.82] [131]. On the other hand, no benefits of vitamin D supplementation were observed in children with mean baseline levels <35 ng/mL [132]. A metanalysis of RTCs concluded that daily or weekly vitamin D supplementation significantly reduces the risk of acute respiratory infections by 19%. Among those with baseline levels <10 ng/mL, daily or weekly doses of vitamin D were associated with a 70% reduction in risk. Even among those with baseline concentrations >10 ng/mL, a significant 25% reduction was observed [133]. A recent update of this metanalysis included 73,398 participants in RTCs (aged 0 to 95 years) and confirmed the protective effect of vitamin D supplementation with 400 to 1,000 IU daily on acute respiratory infection, independent of baseline levels [134]. Bolus doses, however, were not protective [133, 134].
- **COVID-19**
  A link between the COVID-19 and Vitamin D was hypothesized early in the pandemic [135] due to the aforementioned immunomodulatory actions of vitamin D [4]. Since those early days, several studies, mostly observational, cross-sectional, or retrospective, have attempted to elucidate this potentially very relevant relationship. Vitamin D deficiency or even insufficiency, defined as 25(OH) D below 20 ng/mL and 30 ng/mL respectively were associated with an increased risk of SARS-CoV-2 infection with mean vitamin D values lower in subjects who tested positive [136]. Not all published studies are in agreement with these data because others did not find any significant relationship between vitamin D status and predisposition to SARS-CoV-2 infection risk [137].
  Vitamin D deficiency was also reported to correlate with the severity of COVID-19, particularly in the elderly. Besides the frequent finding of hypovitaminosis D in patients with comorbidities such as diabetes mellitus and obesity [105], which are known to increase the severity of COVID-19 [138, 139], it was found that subjects with severe disease were more likely to have vitamin D deficiency and obesity or hyperglycemia [41] as compared to patients with less severe forms of the disease. Nevertheless, even mild vitamin D insufficiency consistently predicts hospitalization and mortality [140]. Moreover, very low vitamin D levels appear to be associated with greater risk for admission to an intensive care unit (ICU) [141] and consequent mortality (50%) [142, 143].
  Only few interventional placebo-controlled prospective randomized studies have been published so far, with contrasting results [143, 144]. Hospitalized COVID-19 patients treated with calcifediol (532 ug on day one plus 266 ug on days 3, 7, 15, and 30) had lower risk (OR 0.13) of ICU admission and mortality (OR 0.21) [145]. However, in another study, a single dose of 200,000 IU vitamin D3 in COVID-19 hospitalized patients did not reduce mortality or time of hospitalization [146]. Future studies are needed to confirm these observations, taking into account possible confounding factors.
  In COVID-19 patients, vitamin D deficiency has been observed also to be associated, with an impaired PTH response, with hypocalcemia [147–151]. COVID-19 related hypocalcemia has been recently identified by several studies, reviews and meta-analyses as a potentially useful biomarker for disease severity and outcome in patients with SARS-CoV-2 infection [152–154]. Therefore, vitamin D supplementation might have a therapeutic role in these patients [6]. Another unresolved issue is related to the role of maintaining adequate 25 OH Vitamin D levels [20] in the post-COVID-19 recovery or persistent phase [155, 156].
  Although not universally accepted guidelines on vitamin D treatment in the prevention of COVID-19 are available [143], it seems reasonable to recommend a goal of 25-OH Vitamin D levels >30 ng/mL. This would include those who are at particularly high risk such as older men with co-morbidities such as diabetes and obesity [105, 157].

## Variability in the form of VD that is needed for specific situations

- **Vitamin D3 and vitamin D2: When and why to use vitamin D3**
  Cutaneous production of cholecalciferol is the dominant vitamin D source in humans. Thus, when vitamin D supplementation is needed, use of cholecalciferol (vitamin D3) rather than ergocalciferol (vitamin D2) is logical. However, it is appropriate to recognize that small concentrations of 25(OH)D2 are present in human population studies [158]. These small concentrations may reflect food sources, as comparable levels are observed in free ranging baboons [159]. Additionally, much higher 25(OH)D2 levels may be observed clinically as ergocalciferol is widely available and highly prescribed in some countries. Thus, it is reasonable to ask whether vitamin D2 and D3 are equivalent.
  Despite both being considered “vitamin D,” subtle physiologic differences do exist, e.g., the affinity of VDBP for vitamin D2 metabolites is less thaìn for vitamin D3 which likely leads to approximately 10% shorter half-life of 25(OH)D2 compared with 25(OH)D3 [160]. Moreover, a systematic review of studies comparing vitamin D2 and vitamin D3 supplementation found cholecalciferol to produce greater increments in circulating total 25(OH)D concentration than does ergocalciferol [161].
  However, others observe no difference leading some guidelines to consider vitamin D2 and D3 as clinically interchangeable [162] echoing guidance of 80 years ago finding equivalency in the treatment of rickets [163]. Thus, ergocalciferol is clearly biologically active. It is appropriate to recognize that provision of high-dose ergocalciferol reduces 25(OH)D3 concentration [164] likely via competition at the 25-hydroxylase level.
  Nonetheless, based upon lower binding to VDBP, shorter half-life and reduced efficacy in raising 25(OH)D, some have recommended that ergocalciferol not be utilized clinically [165]. Of perhaps even greater importance than these physiologic differences are observations that the substantial amounts of both 25(OH)D2 and 25(OH)D3 may lead to erroneous total 25(OH)D measurements [166, 167]. These incorrect results likely reflect major analytic challenges to measurement of total 25(OH)D by immunoassay; namely the antibodies may not detect 25(OH)D2 and 25(OH)D3 equally and/or the proprietary approach used in automated immunoassays to release these vitamin D metabolites may not liberate them equally from VDBP [168]. Based on the above noted physiological differences and analytic challenges to 25(OH)D measurement, recent guidance advises against ergocalciferol use when there is a choice to be made [169].
- **Use of calcidiol (1-alpha analogue) versus calcitriol**
  Calcitriol (1alpha,25-dihydroxyvitamin D) is the most active metabolite of vitamin D. Alfacalcidol is a synthetic calcitriol analogue (1alpha-hydroxyvitamin D) which is 25-hydroxylated in the liver into calcitriol, avoiding the need for enzymatic conversion by the renal 1alpha-hydroxylase enzyme [170]. Not surprisingly, therefore, alfacalcidol increases calcitriol levels independently of renal function. The effects on intestinal calcium absorption and on bone turnover of oral or parenteral administered calcitriol and alfacalcidol are very similar [171]. In general, the use of 1-alpha-hydroxylated forms is hampered by the higher risk of developing hypercalcemia and/or hypercalciuria [172]. However, the biochemical safety of calcitriol and alfacalcidol is similar. Alfacalcidol has been shown to prevent falls and fractures in women with postmenopausal osteoporosis and also in elderly individuals of both sexes [167]. Some possible differences on vertebral fracture risk between calcitriol and alfacalcidol may be related to the populations enrolled in the trials [173].
- **When to use active vitamin D analogues versus 25(OH)D versus vitamin D-alone or in various combinations**
  i. **Hypoparathyroidism**
    Along with oral calcium, active vitamin D is a mainstay of treatment for hypoparathyroidism [174]. Calcidiol can be used as an alternative but in higher doses. The amount of calcitriol needed to control the serum calcium varies among patients (generally from 0.25 to 4 µg/day). Most patients require daily or twice daily calcitriol. Among the factors that can predict a greater need for calcitriol, obesity has recently been recognized [175]. Due to the narrow therapeutic range of calcitriol, added to the lack of effect of PTH on calcium reabsorption on glomerular filtrate, hypercalciuria can be an unwanted adverse effect [176, 177].
    Further metabolism of 25(OH)D to analogues other that the active metabolite may have beneficial systemic effects. Therefore, along with active vitamin D, it is advisable to maintain normal levels of 25OH D in hypoparathyroidism [178]. In those who require large doses of vitamin D orally, the intramuscular route of administration is attractive. This is particularly relevant in those with malabsorption syndromes and hypoparathyroidism.
  ii. **Chronic kidney disease**
    As summarized above, vitamin D administration in CKD is complicated, but a case can be made to administer cholecalciferol in standard doses for bone health and as substrate for eventual local (tissues other than the kidney) production of calcitriol from circulating 25(OH)D. As renal function declines, the kidney’s ability to 1 alpha hydroxylate 25(OH)D decreases, leading to decreased gastrointestinal absorption of calcium and phosphate, although serum phosphate rises because of decreasing renal excretion. For the patient with CKD 5, calcitriol has been the standard form of vitamin D replacement. Other 1 alpha-hydroxylated VD preparations are now available. Based on the KDIGO guideline, in patients with CKD 3 to 5 (but not on dialysis), 1 alpha-hydroxylated analogs and calcitriol are discouraged [99], unless there is severe hyperparathyroidism. Once the CKD 5 patient begins hemodialysis, such analogs or calcitriol should be used in combination with calcimimetics such as cinacalcet to bring serum calcium, phosphate, and parathyroid hormone levels to target ranges. Use of vitamin D analogs has not been shown to be superior to calcitriol [179]. Serial measurements are needed over time with use of phosphate binders and other agents to optimize mineral homeostasis and bone health [180]. Nonetheless, fracture risk is high in CKD 5, and management choices are limited.
  iii. **Severe liver disease**
    In the normal activation sequence of vitamin D, the liver is the source of the 25-hydroxylation step. Thus, it would be expected that patients with severe liver disease, such as cirrhosis, may have vitamin D insufficiency or deficiency. Complicating this is the cause of the liver disease, (biliary cirrhosis versus alcoholic cirrhosis versus cirrhosis from steatohepatitis) which may have different impacts on vitamin D metabolism, VDBP and other proteins. Surprisingly, there is little written about using calcidiol, which is already 25-hydroxylated in patients with severe liver disease. In effect, it bypasses the hepatic step. Calcidiol, available by prescription in Europe, has been used in postmenopausal osteoporosis, instead of cholecalciferol [181]. To our knowledge, there are no trials of calcidiol versus cholecalciferol in patients with severe liver disease. If cholecalciferol is the only available preparation, it may be more important to follow albumin-adjusted or ionized serum calcium in addition to 25(OH)D. Presumably, VDBP can also be affected if there is defective hepatic protein synthetic function. For most patients, cholecalciferol should be adequate, but in severe cases calcidiol when available, or calcitriol, may be necessary. However, there is some evidence that preparations of calcidiol may be more difficult to titrate than cholecalciferol [182].

## Variability in therapeutic regimens to correct vitamin D deficiency

- **Daily dosing vs weekly vs monthly**
  Relatively few studies have evaluated the comparative efficacy and safety of vitamin D treatment in daily, weekly or monthly dosing regimens.
  When the same cumulative dose of vitamin D3 (1,500 IU daily equivalents) was given under daily, weekly or monthly regimens, the mean levels of 25(OH)D over a period of two months were similar with slightly more variability observed when given monthly [183].
  Another prospective randomized open label multicenter 3-month study showed equal efficacy and safety of daily 1,000 IU vs either weekly 7,000 IU or monthly 30,000 IU of vitamin D3 on 64 adults with low 25(OH)D (<20 ng/mL). 25(OH)D values were restored to>20 ng/mL in all groups. Observed increases in 25(OH)D were similar without any statistically significant differences among groups [184].
  In another recent single-center, open-label randomized 12-week study on healthy subjects with low 25 (OH)D (< 20 ng/mL), efficacy and safety of three different schedules (daily, weekly, or bi-weekly) of cholecalciferol (10,000 IU/day for eight weeks followed by 1,000 IU/day for four weeks; 50,000 IU/week for 12 weeks, 100,000 IU/every other week for 12 weeks; total cumulative doses 588,000 IU, 600,000 IU, 600,000 IU, respectively) were tested. All subjects rapidly and safely normalized vitamin D with similar peak 25(OH)D serum levels (81.0 ± 15.0, 63.6 ± 7.9 and 59.4 ± 12 ng/mL respectively) [185].
- **When to use oral versus intramuscular formulations**
  Increases in serum 25(OH)D levels have been compared after either oral or intramuscular administration of the same single dose (300,000 IU) of cholecalciferol or ergosterol in elderly subjects with hypovitaminosis D [186]. Interestingly, the short term (1-month) increase in serum 25(OH)D was about three times with oral vs intramuscular cholecalciferol (peaking at 47.8 ± 7.3 ng/mL vs 15.9 ± 11.3 ng/mL respectively). Similar data, although at lower peaks, were observed with ergosterol. In the longer term (> 2 months) these differences are no longer observed. Therefore, in the general population or when a rapid normalization of vitamin D is needed, the oral route is preferable for loading whereas for maintenance treatment the two routes of administration seem equivalent [171]. Parenteral route of vitamin D administration has been shown to be effective and safe in patients with hypovitaminosis D caused by severe intestinal malabsorption [186]. The term malabsorption can include a large number of disorders, including IBD, pancreatic insufficiency, short-bowel syndrome, gluten enteropathy, post-bariatric surgery, and any need to total parenteral nutrition. Therefore, in these settings, intramuscular vitamin D may be the treatment of choice.

## Future perspectives and conclusions

Vitamin D controls not only skeletal homeostasis but also has a role in many extra-skeletal tissues and organs [4]. A main aim of the Conference was to address the question whether a standard approach to vitamin D supplementation applies equally to all diseases in which vitamin D inadequacy might be implicated. . More attractive is the concept of a tailored approach to vitamin D based on the specific mechanisms underlying vitamin D deficiency in the different diseases. It is apparent that desirable levels of vitamin D and the amount of vitamin D needed to reach such levels will vary depending upon the disease and the organ systems involved. The specific situations where such a tailored approach may be indicated includes obesity, glucocorticoid therapy, malabsorption syndromes, osteoporosis, diabetes mellitus, cancer, infectious diseases, and SARS-Cov-2 infection. Finally, whereas oral cholecalciferol appears to be the preferred choice for vitamin D supplementation in the general population and in most clinical conditions, active vitamin D analogs may be indicated in patients with hypoparathyroidism and severe kidney and liver insufficiency. Parenteral vitamin D administration could have a place in patients affected by malabsorption.

Given the wide variability in vitamin D needs as a function of disease state, our conclusions support the concept that specific guidelines for vitamin D desired levels and doses might be useful to develop for each of the conditions known to affect vitamin D metabolism and in which hypovitaminosis D plays a clinically relevant role.
